# Development and implementation of a nutrition education programme for school-going adolescents in the context of double burden of malnutrition: a narrative essay

**DOI:** 10.11604/pamj.2024.47.40.42456

**Published:** 2024-02-01

**Authors:** Joelle Laure Sobngwi-Tambekou, Magellan Guewo-Fokeng, Jean Claude Katte, Diane Dione Ekwoge, Liliane Kamdem, Leopold Fezeu, Eugene Sobngwi

**Affiliations:** 1 RSD Institute, Yaoundé, Cameroon,; 2 Faculty of Medicine and Biomedical Sciences, University of Yaoundé, Yaoundé, Cameroon

**Keywords:** Nutrition education, adolescent, school, healthy diet, physical activity

## Abstract

The early prevention of non-communicable diseases in Cameroon schools program was initiated in 2018 to address the alarming trend of obesity among adolescents through a nutrition education intervention aimed at increasing knowledge on nutrition and the benefits of healthy eating and physical activity. The program included: school surveys to document eating habits and health-risky behaviors in students, the development of a training curriculum, training and sensitization sessions for school staff, school vendors and students, and advocacy meetings with parliamentarians and mayors. We carried out a quasi-experimental study to assess the effect of the intervention on the student's knowledge and eating behavior three months after the training sessions. We compared the knowledge of a sample of students from five schools that were part of the program (IG) to that of students that were not (CG). The mean (±SD) score was 14.4/20 (±2.1) and 9.7/20 (±2.7) for IG and CG, respectively (p<0.001). Those who scored above 12/20 accounted for 89.8% of IG vs 23.8% of CG (p<0.001). Other significant achievements of this program are the amendment of the National School Hygiene Policy to include compulsory training in food hygiene and nutrition education for school canteen vendors and the integration of nutrition education sensitization sessions into the routine activities of school healthcare. The study showed that a well-structured multi-sectoral nutritional education program could be the bedrock to improve healthy nutrition among adolescents, thereby serving as a vehicle for non-communicable disease prevention.

## Essay

Strong scientific evidence highlights the dramatic shift of diet and physical activity patterns in developing countries characterized by the progressive replacement of traditional diets rich in fiber and cereals by Western diets rich in fat and added sugar, meat, and sedentary lifestyles. This so-called nutrition transition, occurring in a context of rapid economic development and globalization, is coupled with a double disease burden characterized by a slow decline in malnutrition and infectious diseases and a rapid increase in obesity and non-communicable diseases [1,2]. For example, in Cameroon, the prevalence of underweight among adolescent girls has been stagnant at around 10% since 2004 [3], while the prevalence of diabetes in the adult population over the same period has tripled from 2% to 6% [4]. Rapid and uncontrolled urbanization, conflicts, and social unrest further increase food insecurity in the most vulnerable populations, thus promoting erratic nutrition habits [5]. While significant efforts are being made to curb malnutrition, particularly undernutrition, little or no attention and investments are committed in many African countries to address overweight and obesity, particularly among children and adolescents [6,7].

Childhood and adolescent overweight and obesity are a matter of concern because of the immediate and long-term consequences on health. Childhood obesity is associated with the early onset of cardiovascular risk factors, including hypertension and dysglycemia, potential risk factors for the onset of chronic diseases, and premature mortality in adulthood if it persists [8,9]. Overweight and obesity in children and adolescents are steadily increasing in most Low- and Middle-Income Countries (LMICs), particularly in sub-Saharan Africa (SSA). Systematic reviews of studies conducted between 1964 and 2019 on obesity in school-aged children and adolescents in SSA countries show a prevalence between 9.5 and 11.5% for overweight and 2.5 and 8.6% for obesity, depending on the cut-off points used [2,10,11]. Although these proportions are lower than those observed in developed countries, they are considered alarming due to the rapidly increasing trends.

Scientific literature presents several examples of interventions to prevent and address overweight and obesity in children and adolescents [12-21]. Most of these interventions are implemented in schools. Schools are one of the most appropriate sites for health education interventions targeting children for at least two reasons: firstly, schools are an organized setting that brings together as many children as possible for more than half of the day and, secondly, teachers are important facilitators for the implementation of interventions targeting children [18]. Their ownership and involvement guarantee the sustainability of these interventions. In addition, school attendance rates favor this approach to reach the maximum number of children and adolescents. While several interventions to prevent obesity among children and adolescents have been carried out in schools and communities in developed countries, data on school-based interventions in Africa are relatively scarce. The only published systematic review of school-based interventions targeting nutrition, physical activity, and body weight status of African children identified nine interventions conducted in two African countries: South Africa and Tunisia [13]. Usually, school-based health interventions implemented in LMICs mainly focus on reproductive health, personal hygiene, HIV/AIDS prevention, substance abuse, and violence prevention, with little attention paid to the growing epidemic of overweight and obesity.

In Cameroon, the prevalence of overweight in children under five years has almost doubled over the last 15 years, rising from 6% in 2011 to 11% in 2018. According to data from the latest Demographic and Health Survey conducted in 2018, about one in five adolescent girls is overweight or obese, with urban settings being the most affected [3,22]. In this context, in partnership with the Ministry of Secondary Education, we decided to develop and pilot the nutrition education program and physical activity promotion in schools in the central region of Cameroon. Specifically, the aims of the "Early Prevention of Non-Communicable Diseases (EP-NCDs) in Cameroon Schools" program were to 1) promote healthy eating habits and physical activity in adolescents to prevent NCDs by developing and implementing a culturally appropriate nutrition education and physical activity program, and 2) contribute to high-level advocacy for a sustainable healthy diet and physical activity promotion in children and adolescents. This paper describes the design, implementation, and key lessons learned throughout the program.

**Figure 1:**
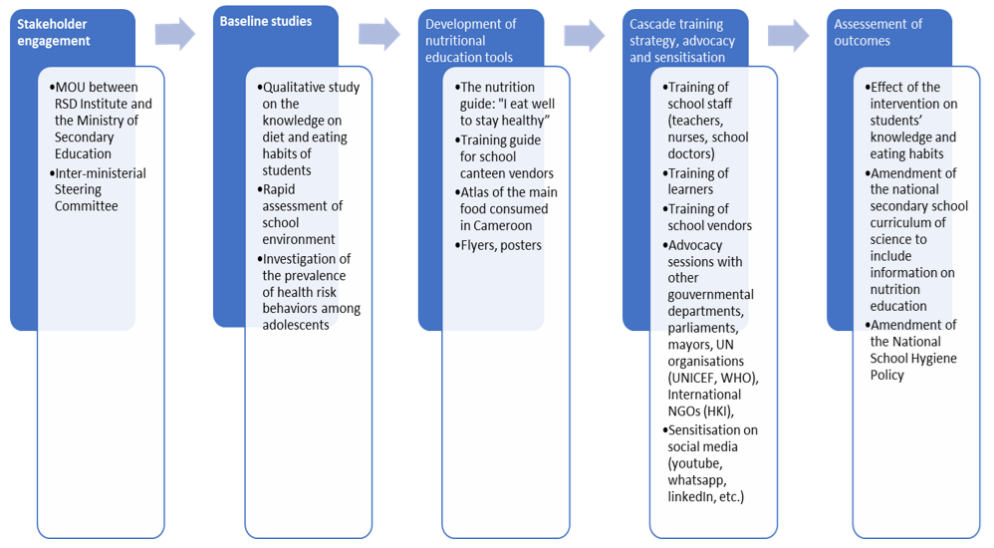
key activities implemented during the "early prevention of non-communicable diseases in Cameroon schools"

**Designing and implementing the "Early Prevention of Non-Communicable Diseases in Cameroon schools" program:** the program was developed using extensive formative research to assist in designing and implementing the intervention. Thus, qualitative, and quantitative assessments were performed within the target community before the implementation phase to identify their interests, needs, and expectations to develop a tailored, contextualized, and meaningful intervention [23]. Figure 1 summarizes the key activities performed during the implementation of the program.

**Stakeholder engagement:** the effective kick-off of the project was marked by the signing of a memorandum of understanding (MOU) in April 2018 between the RSD Institute and the Ministry of Secondary Education for developing and implementing a nutrition education program in schools to prevent NCDs. An inter-ministerial steering committee was then established to monitor the program. This committee was made up of representatives from the Ministry of Secondary Education, the Ministry of Public Health, specifically the National Diabetes Program, and members of the research team. Throughout the design and implementation phases of the program, several meetings were held with these key stakeholders to refine the planning and implementation strategies of the program. These meetings were crucial to ensure the buy-in and ownership of the program by the key stakeholders, the identification of the real needs of the targeted populations, and the adequacy of the proposed interventions.

**Baseline assessments:** a qualitative assessment of the knowledge of diet and eating habits of secondary school students: This assessment aimed to examine the students' eating habits, their knowledge of the relationship between diet, physical activity, and health, and the determinants of their food choices. Focus group discussions (FGDs), gathering ten boys and girls aged 14 to 16 years each, were organized in four schools in Yaoundé, two in urban areas and two in rural areas. Most of the students reported that they usually had breakfast at school. Breakfast consisted mainly of foods made of refined cereal flour, rich in fat and sugar (doughnuts). The consumption of fruits and vegetables was insufficient, limited to once or twice a week. The students admitted that they regularly (almost every day) consume sweetened-carbonated drinks or sweetened drinks made from local fruits.

According to these students, the factors that determine their food choices are the palatability of food, family eating habits, and the cost and availability of food. The diet of many students was mainly traditional and dependent on the region of origin of the parents. According to most of the informants, a "healthy diet" should be diversified with "a lot" of fruits and vegetables, little salt, little fat, and little sugar. All students interviewed were aware of the health consequences of poor diet. Most informants at the end of the discussion perceived their diet as poor quality. Informants emphasized the need to improve the quality and variety of food available in school canteens. The rapid assessment of the school environment: The policy, physical, and cultural environments of the four selected secondary schools were assessed using an adaptation of the observation grid provided by Saluja *et al*. [24]. The policy assessment highlighted the existence of national guidelines governing the selection and supervision of school vendors and food and canteen hygiene. This policy was implemented in all the schools surveyed. However, none of the schools had guidelines on the food quality provided in the canteen. The schools also had education programs related to alcohol and tobacco consumption, with guidelines prohibiting the sale of these items in and around the school. Still, none offered a nutrition education program for children, school staff, or parents.

Three of the four schools had a clean canteen, but none had piped water or facilities for washing hands or utensils. Drinking water points were present in three out of four schools. The meals sold in the canteen were primarily high in fat, sugar, and salt (fried food, dishes with sauce, doughnuts, pastries). This observation motivated the inclusion of a training workshop for school canteen vendors in food hygiene and healthy cooking methods as a component of the intervention. No posters promoting healthy eating were in these schools, but signages against alcohol and tobacco consumption were displayed in each school. One of the schools did not have a dedicated area for physical activity. In the area of culture, none of the schools had ever organized an event to promote a healthy and balanced diet. In contrast, all the schools organized annual events related to physical activity (mainly tournaments and several different sporting competitions).

Investigating the prevalence of health risk behaviors among adolescents: We adapted the Global School-Based Student Health Survey (GSHS) questionnaire developed by WHO and CDC Atlanta [25]. A joint team of the Ministry of Secondary Education and RSD Institute conducted data collection. The Yaoundé-GSHS (Y-GSHS) was implemented in 10 schools in the city, selected from urban centers and rural suburbs. The adapted GSHS questionnaire included information on dietary behaviors, smoking, alcohol and drug use, hygiene, violence and unintentional injuries, mental health, sexual behaviors, and physical activity. A total of 3,241 students aged 12-18 participated in the survey (54.4% male and 45.6% female). Of these, 19.6 % were overweight, and 0.9% were obese. More than a quarter of participants (28%) poorly perceived their weight. Regarding eating habits, 38.5%, and 46.9% reported that they consumed fatty food and food rich in salt, respectively, at least once a day during the preceding 30 days, and some (8.1%) reported frequently skipping breakfast during the same period; 31.7% reported drinking at least one sweetened soft drink every day. The majority (64.6%) declared not being made aware of the benefits of a healthy and balanced diet through the school curriculum since the beginning of the school year. Most students (69.3%) reported having ever consumed alcohol, and more than 40% consumed alcohol regularly. Almost half of the students (40.7%) had not participated in physical education and sports activities one month after the beginning of the school year.

**Survey of commonly consumed foods by the main agro-ecological regions of Cameroon:** we, first of all, started by surveying the different foodstuffs and meals commonly consumed in Cameroon. The survey used a semi-structured questionnaire and respondents were students, parents, food vendors, and nutritional professionals from the different regions of the country and a rigorous literature review. We used data from laboratory reports and published literature to determine the values corresponding to the quantity of a nutrient in each food or dish presented. Food values correspond to the average values of the collected compositional data.

**Development of nutrition education tools:** insights from the baseline studies and discussions with the different stakeholders contributed to finalizing the design of the intervention and the messages to be incorporated into the training and communication tools. The focus was to provide adolescents and the educational community with nutritional information, including traditional foods and culinary knowledge, to ensure their appropriation and use. A diverse group of specialists contributed to developing the educational tools: social scientists, clinical and basic nutrition specialists, dieticians, endocrinologists, public health physicians, pedagogic inspectors, secondary school teachers, and graphic designers. The nutritional guide: "I eat well to stay healthy": We developed a nutrition guide to train teachers, school health nurses, and other educational community members. This guide presents the basic concepts and benefits of a healthy and balanced diet according to the Competency-Based Approach (CBA) principles. Competency-Based Approach (CBA) emphasizes strengthening the lessons' practical, operational dimension [26]. This guide comprises nine (9) modules and exercises that allow the facilitator to assess the trainees' knowledge at the end of the training. The modules include information on the different types of nutrients and their role, the importance of eating healthy and balanced, the different kinds of food and cooking methods, the importance of physical activity for health, and a typical day's diet. The guide was designed to be used in bulk or as separate lesson items. The modules in the guide were construed to be taught as part of the secondary school science courses either during extra-curricular activity time slots or as a separate food and nutrition subject.

Facilitator's guide for trainers (teachers, peer educators): the facilitator's guide was designed to be used with the Nutritional Guide: "I eat well to stay healthy." This document provides a plan for organizing discussion sessions with adolescents and instructions on presenting each module and using the different tools developed to facilitate a nutrition education session. Training guide for school canteen vendors: schools usually have canteens where licensed vendors offer a variety of foods. To improve the nutritional quality of the food in school canteens, we developed a manual to train vendors in the basic principles of a healthy and balanced diet, healthy cooking methods, and food hygiene. It is anticipated that this training will become mandatory as hygiene courses are before the accreditation of vendors. Atlas of staple foods in Cameroon and the "AfricaFood Atlas" app: At the start of the project, there was no reference document in Cameroon presenting the leading food consumed in the country's agro-ecological and cultural regions. Information on the composition of different foods is the basis for nutritional advice for preventing and managing nutrition-related diseases. Therefore, we considered it essential to combine the various training materials with an information document on the energy values of the various foods and dishes eaten in the country. We compiled the different foods and dishes consumed in Cameroon from our baseline assessment survey into an atlas with their average caloric composition.

**Figure 2:**
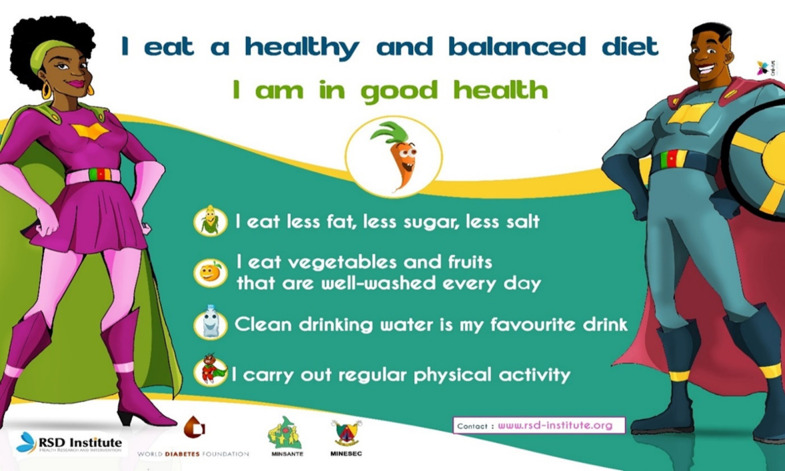
I eat a healthy and balanced diet, I am in good health

To reach the widest audience possible, we also developed an android version of the Atlas. This application - AfricaFood Atlas - also allows the users to compose their meals according to their caloric values. The "AfricaFood Atlas" app is available on Google Play store®. The sensitization tools for nutritional education: We developed posters and leaflets taking up key lessons of the nutritional guide to explain through simple and entertaining messages the importance of a healthy diet and the advantages and disadvantages of the different cooking methods, with the help of young local computer graphics designers. The graphic design was based on cartoon illustrations of the various foods to arouse the curiosity of young people, encourage them to read the leaflets, and make it easier for them to grasp the messages conveyed. Figure 2 shows the poster "I eat a healthy and balanced diet; I am in good health". This poster summarizes the four essential messages of the entire content of the program: 1) "I eat less fat, less salt, less sugars", 2) "I eat vegetables and fruits that are well washed every day", 3) "Clean drinking water is my favorite drink", 4) "I carry out regular physical activity". Local graphic designers proposed young and fit male and female superheroes that teenagers can identify with to facilitate the appropriation of the messages.

**Figure 3:**
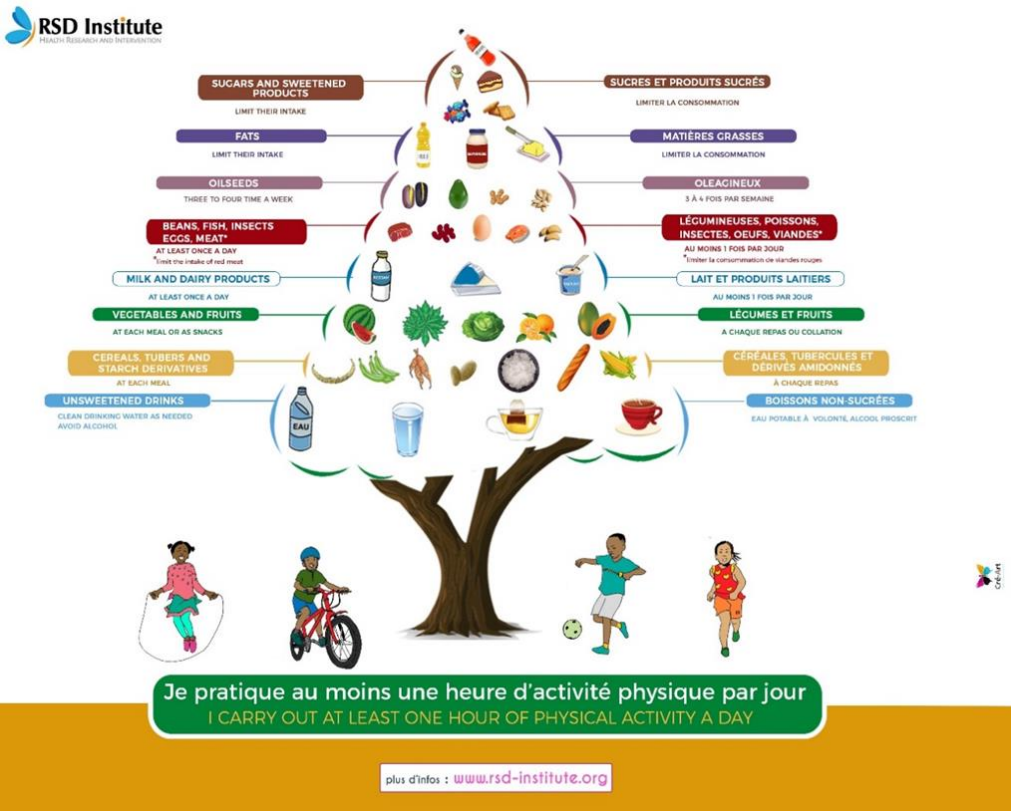
the food pyramid

**Figure 4:**
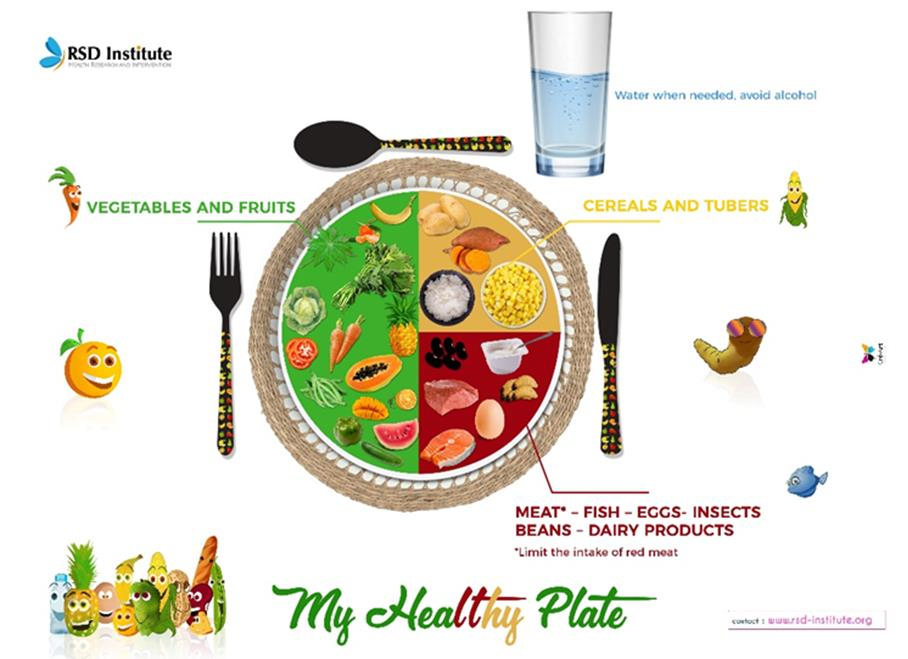
my healthy plate

Figure 3 shows the poster "The food pyramid". This simple visual decision-making tool presents a hierarchy of food types according to the "desirable" frequency with which they can be consumed. The aim is to help users refine their food choices as part of a balanced diet. Our food pyramid used the "nutrition tree" model, presenting the different types of local foods easily accessible to most of the population. Local sources of animal protein (traditional dairy foods, insects, and larvae) and tropical fruits not included in existing food pyramids are represented on this nutrition tree. Specific references to adolescents are retained on the pyramid, such as the prohibition of alcohol. Adolescents engaged in physical activity are illustrated under the "tree" to reinforce the promotion of physical activity. Figure 4 shows the poster "Healthy Plate." This visual decision-making tool shows how a plate should be composed for a balanced meal: half of the plate is made up of fruits and vegetables, a quarter of vegetal and/or animal proteins, and the other quarter of carbohydrates (starchy foods). Vegetables and other local protein sources are prominently featured on the plate to encourage adolescents to prioritize the most accessible foods in their environment. One specificity of the proposed healthy plate is that it accounts for the cultural and socio-economic contexts of most communities in Cameroon that have one-course meal practices. The validation and translation of the curriculum documents: The content of all these documents was validated by a working group composed of experts from the Ministry of Secondary Education, the Ministry of Public Health, RSD Institute, Teachers, and the end users, students. The validation committee proposed that the modules of the nutrition guide should be integrated into the national science curriculum for secondary schools. These documents were initially developed in French, then translated into English, and back-translated into French by independent experts who did not develop the original texts.

**The cascade training strategy, high-level advocacy, and raising of awareness campaign:** the cascade training began with "Training of Trainers" sessions. The trainers were pedagogical inspectors, medical-school inspectors, and school healthcare workers (nurses and doctors). They, in turn, trained other teachers (sciences teachers and teachers in charge of extra-curricular activities) and school nurses. In schools, teachers were responsible for conducting nutrition education sessions of at least 30 minutes with students during science classes, in students' clubs, and during school events. A total of six (6) sessions were needed to cover the whole program. These sessions included a practical phase as far as possible. Overall, 379 teachers and 24,172 students have been trained in 47 schools between November 2020 and February 2021.

School medical doctors took the opportunity of the students' annual medical check-ups to raise awareness of the benefits of a balanced and healthy diet. Before the individual medical visits began, the school medical doctors organized health talks on the benefits of physical activity and a healthy diet. A total of 242,014 students were sensitized in 537 secondary schools in the central region of the country. The school nurses were responsible for training the accredited vendors in their respective schools. The training aimed to improve food hygiene, diversity, and quality in school canteens. Tips on how to cook low-cost healthy and tasty food were shared with the vendors. More than 520 vendors and nearly 400 other administrative staff have been trained. At the end of the training, several vendors reported that they had learned how to cook with less fat and how to ensure the food and the environment were hygienic. The Minister of Secondary Education then decided that the training of vendors should become a prerequisite certification for any vendor willing to be admitted to selling in a secondary school canteen. The National School Hygiene Policy was amended to take account of this new requirement. We implemented high-level advocacy meetings with different groups of decision-makers. We shared our experience and discussed the importance of school nutrition education in a plenary session of the parliament and during a meeting of the mayors of the country's main cities. Meetings were also organized with different members of the government (Ministry of Health, Ministry of Secondary Education, Ministry of Basic Education, Ministry of Public Service - members of the inter-ministerial committee for the fight against malnutrition), United Nations Organizations (WHO, UNICEF) and international non-governmental organizations involved in nutrition and adolescent health (Hellen Keller International). These different meetings were opportunities to discuss the challenges of the double burden of malnutrition in the population, specifically among adolescents, and to reflect on potential interventions.

We also continued to raise awareness of healthy and balanced diets through social networks (YouTube, Facebook, Twitter, LinkedIn, Instagram, and Whatsapp) with weekly posts on the different types of food and their health benefits, cooking tips to get the best out of food and physical activity. Three (3) months after the training sessions in the schools, we piloted a quasi-experimental study to assess the effect of the training on the student's knowledge and eating behavior. We assessed and compared the knowledge of a sample of 500 students from three city high schools participating in the program (intervention group - IG) with that of 500 students from three city high schools not involved in the program (control group - CG). The schools were paired according to their location (urban/rural). We assessed the students' knowledge using a 20-item questionnaire on the program's key messages. The mean score (±SD) in the two groups was 14.4 (± 2.1) and 9.7 (± 2.7) for IG and CG, respectively (p=0.001)). Those who scored above 12/20 accounted for 89.8% of IG compared to 23.8% of CG (p=0.001). A detailed analysis of the responses to the different questions showed that IG students were significantly better at answering questions on the composition of a healthy meal (85.4% vs. 37.2%); the characteristics of different types of food, the cooking methods, and storing of food. For example, 62.6% of students in the IG versus 10.0% of the CG gave the correct answer to the question "What is the highest energy-yielding nutrient?" and 81.2% in the IG knew that cooking oil should not be re-used for frying it smokes, foams, or smells versus only 40.2% in the CG (p=0.001). Similarly, 85.4% of students in the IG compared to 51.4% in the CG (p=0.001) knew that the use of plastic bags for storing hot food is prohibited. We assessed eating behavior using the 24-hour dietary recall. The results revealed a higher consumption of fried foods and sweets and a lower intake of fruits and vegetables in the CG group. However, these results were not significant. Overall, these results showed a significant improvement in the knowledge of the students who participated in the nutrition education sessions. They also allowed us to identify other interventions that future campaigns should focus on to improve adolescents' eating habits significantly.

**Revision of the health policy and the secondary school science curriculum:** in Cameroon, there is no national school feeding policy and strategy. The only policy related to school feeding in the country is an administrative circular note from the Minister of Secondary Education (Circular N°27/10 MINESEC/SG/DRH/SDSSAPPS), on the organization of the national hygiene policy in public and private secondary schools. This administrative circular note, which dates from 2010, specifies the rules of hygiene and sanitation as well as the conditions to be met by vendors of school canteens. As part of this program, we have requested the revision of this circular. To this end, a workshop was organized with the School Medical Inspectors of the country's ten regions to review and update the circular note. At the end of this workshop, the circular note was updated with vendor information, food, and environmental hygiene, drinking water supply, creation and use of latrines, garbage collection and management, and supervision of the implementation of the policy. Specific recommendations on hygiene procedures in the context of an epidemic (e.g. the Covid-19 pandemic) were also included in the circular note. Finally, the Minister of Secondary Education instructed that the validation of the sales training developed by the program be included among the conditions to be met to be admitted as a food vendor in any secondary school. As a continuation of this program, a working group of teachers (Regional Science Coordinating Inspectors), School Medical Inspectors, experts in nutrition, and public health and student representatives was set up to validate the nutrition education content in the secondary school program. The content of all secondary school classes (French and English sub-systems) was reviewed by this working group, the basic principles of nutrition education were updated in the scientific content from year 7 to upper-sixth classes, and thematic cards were developed to help teachers in the preparation of nutrition education lessons.

**Public health relevance of this program to the local context:** this paper describes developing and implementing the "Early Prevention of Non-Communicable Diseases (EP-NCDs) in Cameroon Schools" program. It emphasizes the importance of multisectoral action in delivering any public health intervention. Major stakeholders were experts from non-governmental institutions, the Ministry of Secondary Education, the Ministry of Public Health, and several other independent experts on nutritional education. As part of this program, a global school health survey was conducted amongst 3241 adolescents aged 12 - 18 years, assessing risky behavioral patterns. Furthermore, several nutritional education tools (a nutritional guide, facilitator's manual, comprehensive food atlas, posters, flyers) and a nutritional education training program were piloted. At the end of this pilot program, some significant policy achievements include 1) the revision of the National School Hygiene Policy, which makes it compulsory for food vendors to be trained in food hygiene and nutrition education before being allowed to work in school canteens, and 2) the revision of nutrition education information and material in the science program and 3) the integration of nutrition education sensitization sessions into the routine activities of school healthcare. Several factors have contributed to the success of this program. First, the formative research and the multisectoral approach (staff and experts from the Ministries of Secondary Education and Public Health) throughout the implementation process contributed to ensuring the ownership and acceptance of the program and the design of context-appropriate and culturally sensitive interventions. Secondly, teachers and students collaborated with young local graphic designers to develop the communication and sensitization materials. This collaboration resulted in the production of attractive and user-friendly educational posters and flyers. Third, advocacy and partnership with other organizations contributed to the buy-in and involvement of institutions such as UNICEF (Cameroon office) in the science curriculum revision process. Fourth, integrating educational talks on nutrition and physical activity during students' annual medical check-ups seemed a very efficient model to raise awareness of the health benefits of a balanced diet and physical activity among more than 200,000 adolescents.

The revision and implementation of the "National Secondary School Hygiene Policy" to recommend the training of school canteen vendors in food hygiene and healthy diet, as a prerequisite for admission to sell in a school canteen, will contribute to improving adolescents' exposure to balanced and healthy food choices in schools. Also, including updated information and material for nutrition education in the science curriculum may be a sustainable means to improve the dissemination of adequate and context-adapted knowledge on nutritional education and healthy food choices in Cameroon. However, long-term research will be needed to evaluate this measure. There is growing evidence to suggest that strategies that target change at the levels of policy and macro-environment are more cost-effective with more significant potential for long-term sustainability than those that target change at the individual level [27,28]. Systematic reviews of intervention programs for childhood obesity prevention via schools in developed countries reported that programs promoting relevant policy and environmental changes are more effective [12,15]. The impact of these guidelines on adolescent eating behaviors and health will depend on how rigorously they are implemented. In a rural, low-income South Africa, de Villiers *et al*. implemented an intervention that included the school nutrition policy, physical activity, sports environment, staff health, and chronic disease and diabetes awareness. However, the intervention did not significantly improve children's diet quality because of insufficient rigor in its implementation [29,30]. This program required tremendous effort to set up due to the scarcity of data on similar programs in SSA during its development. There are seldom reports of such primary prevention programs; therefore, there is limited opportunity to compare and anticipate the program's performance [13]. One other major challenge in implementing such intervention is the context of the double burden of malnutrition, which means the coexistence of undernutrition and overnutrition, especially in a setting of wide socio-economic disparities between school attendees. The formatting of sessions and supporting documentation needed to be considered carefully to allow appropriate behavioral influences without discrimination.

Teaching nutrition education modules in class was particularly challenging due to time constraints. Science teachers could only devote one or two sessions of one hour each per class to nutrition education. As a result, only the key modules were covered. This situation also prevented teachers from administering pre-and post-tests, which made it impossible to assess changes in knowledge among the students who received the intervention. In addition to the lessons during biology classes, talks with the students would be carried out in clubs during the extra-curricular activities. Unfortunately, some schools canceled these activities due to the COVID-19 pandemic. Despite all these challenges, the knowledge assessment significantly differed between the intervention and control students. Significant improvements in nutrition knowledge have been reported in several other school-based nutrition education and physical activity interventions among adolescents in Sub-Saharan African countries [31-34]. The use of visuals such as the "healthy plate," which illustrates the composition of a balanced plate, proved to be particularly effective. One of the most discriminating questions between pupils who had or had not received the intervention was related to the composition of a healthy plate. The 24-H dietary recall revealed no difference in the students' eating habits from both groups despite improved knowledge. Several factors may explain this result. The duration of the intervention: according to the literature, interventions with longer duration (at least six months) are more likely to influence adolescent eating behaviors and anthropometric parameters [21]. In this study, the limited duration of the education sessions may have caused the trainers to overlook essential topics or to skip the practical phase that would have allowed the students to absorb the information better. In addition, the program did not include booster sessions, which could provide additional motivation and increased self-efficacy, leading the adolescents towards healthier diet practices. Similar results have been documented in several other studies [29,35]. Although the program incorporated an environmental component with vendor training and school hygiene policy changes, involving families, specifically parents/caregivers, could have improved the program's effectiveness [18,36].

**Implications for research and practice:** the rising rates of overweight and obesity among children and adolescents in sub-Saharan Africa is a cause for concern given their associated short and long-term social and health consequences. Interventions targeting this specific population are urgently needed to reduce these health risks. Therefore, the "Early Prevention of Non-Communicable Diseases (EP-NCDs) in Cameroon Schools" program has important implications for future adolescent nutrition-related interventions. This is one of the few nutritional education programs conducted in sub-Saharan Africa targeting adolescents. The baseline qualitative and quantitative analysis conducted in secondary schools helped identify risky eating patterns and unhealthy food available at different school canteens. This program has been piloted in the capital city of Yaoundé with schools selected from both urban and rural settings. More research should be encouraged in other regions of the country to provide decision-makers with a globalist view to inform the formulation of national policies. We found that school canteens and food vendors were a crucial element in the chain of nutritional food choices for secondary school students. One of this program's major educational and training arms targeted school food vendors for appropriate training and accreditation, which has now been formally included in the revised national school health and hygiene policy document. Long-term evaluation of this measure on the overall food availability and quality in school canteens will be needed. This nutritional education program geared the revision of the national school health and hygiene policy (circular note) with the revision of the science curriculum for nutrition taught in secondary schools. Efficient and sustainable policies such as a National School Nutrition Program with evidence-based and culturally sensitive guidelines for nutrition in schools should be developed to improve healthy eating habits in children and adolescents. Efforts should be made to engage more partners to ensure comprehensive nutrition education programs with strong community participation and ownership are piloted and expanded throughout the country. The design and implementation of this "Early Prevention of Non-Communicable Diseases (EP-NCDs) in Cameroon Schools" program may serve as a starting point to develop a fuller and comprehensive National School Health Policy to be implemented in all the secondary schools in the nation.
